# The relationship between pain and depression and anxiety in patients with inflammatory arthritis: a systematic review protocol

**DOI:** 10.1007/s00296-023-05450-y

**Published:** 2023-09-12

**Authors:** Natasha Cox, Ashley Hawarden, Ram Bajpai, Saeed Farooq, Helen Twohig, Sara Muller, Ian C. Scott

**Affiliations:** 1 Primary Care Centre Versus Arthritis, School of Medicine, Keele University, Keele, UK; 2 Haywood Academic Rheumatology Centre, Haywood Hospital, Midlands Partnership University NHS Foundation Trust, High Lane, Burslem, Staffordshire, UK; 3 Research and Innovation Department, Midlands Partnership University NHS Foundation Trust, Staffordshire, UK

**Keywords:** Arthritis, Rheumatoid, Spondyloarthropathies, Pain, Depression, Anxiety, Systematic review

## Abstract

Pain is a major challenge for patients with inflammatory arthritis (IA). Depression and anxiety are common comorbidities in IA, associating with worse outcomes. How they relate to pain is uncertain, with existing systematic reviews (a) mainly considering cross-sectional studies, (b) focusing on the relationship between pain and mental health in the context of disease activity/quality of life, and (c) not specifically considering the impact of treating depression/anxiety on pain. This PROSPERO-registered (CRD42023411823) systematic review will address this knowledge-gap by synthesizing evidence to summarise the associations (and potential mediators) between pain and depression/anxiety and evaluate the impact of treating co-morbid depression/anxiety on pain in IA. Relevant databases will be searched, articles screened and their quality appraised (using Joanna Briggs Institute critical appraisal tools) by two reviewers. Eligible studies will include adults with rheumatoid arthritis or spondyloarthritis, be a clinical trial or observational study, and either (a) report the relationship between pain and depression/anxiety (observational studies/baseline trials), or (b) randomise participants to a pharmacological or psychological treatment to manage depression/anxiety with a pain outcome as an endpoint (trials). To synthesise data on the association between pain and depression/anxiety, where available adjusted coefficients from regression models will be pooled in a random-effects meta-analysis. A synthesis without meta-analysis will summarise mediators. To evaluate the impact of treating depression/anxiety on pain, endpoint mean differences between treatment arms will be combined in a random-effects meta-analysis. Through understanding how depression/anxiety contribute to pain in IA, our review has the potential to help optimise approaches to IA pain.

**Supplementary Information:**

The online version contains supplementary material available at 10.1007/s00296-023-05450-y.

## Background and rationale

Pain is a major challenge for patients with inflammatory arthritis (IA), with many reporting moderate/severe pain [1], and rating pain as the health area they most wish improved [2]. Despite treat-to-target strategies transforming many clinical outcomes, over 10% of patients with rheumatoid arthritis (RA) in remission/low disease activity experience moderate/high pain [3]. Consequently, understanding which non-disease activity focused approaches best improve IA pain is crucial.

Depression and anxiety are prevalent within the general population, and substantially commoner in patients with IA [4, 5]. Co-morbid depression and anxiety in IA are associated with increased mortality, disability, and disease activity [6]. Whilst often considered that depression associates with worse pain in IA [7], the evidence-base appears conflicting; a meta-analysis by Zhang et al. reported depression was not associated with pain intensity visual analogue scale (VAS) scores in RA, but was associated with short-form (SF)-36 bodily pain scores [8]. This review, however, only considered cross-sectional data. Whilst a systematic review by Rathbun et al. did consider this relationship within longitudinal studies, it was conducted a decade ago and identified only one relevant study, reporting an association between baseline depression and increasing pain VAS scores [9]. Since its publication further longitudinal studies support the perspective that depression in IA associates with more pain [10], and a bidirectional relationship may exist [11].

The association between anxiety and pain in IA has only been considered within systematic reviews examining anxiety’s relationship with disease activity/quality of life. Within this context, Machin et al. identified four relevant cross-sectional studies, reporting significant associations/correlations between pain and anxiety in RA [12], and Zhao et al. identified two studies in psoriatic arthritis (PsA), reporting higher disease activity and pain in those with co-morbid anxiety/depression [5]. As these reviews required studies to have evaluated disease activity/quality of life, they likely missed some pain-relevant studies.

The impact of treating co-morbid depression and anxiety on pain in IA is also unclear. An umbrella review reported that psychological interventions in RA provided small but statistically significant improvements in pain scores post-intervention [13], and a 2011 Cochrane review reported insufficient data (from eight randomised controlled trials) to draw conclusions on the efficacy of antidepressants for pain management in RA [14]. However, these reviews did not specifically focus on the effects of treating depression/anxiety on IA pain, with individual trials suggesting this may be beneficial [15].

This systematic review will address the above evidence-gaps, by providing a comprehensive evidence synthesis of studies examining the relationship between pain and depression/anxiety in patients with IA.

## Objectives

This review has three inter-related objectives in patients with IA: to (1) define the associations between pain and depression and anxiety; (2) describe the potential mediators of these associations; (3) evaluate the impact of treating depression and anxiety on pain.

## Methods

### Registration

**Fig. 1:**
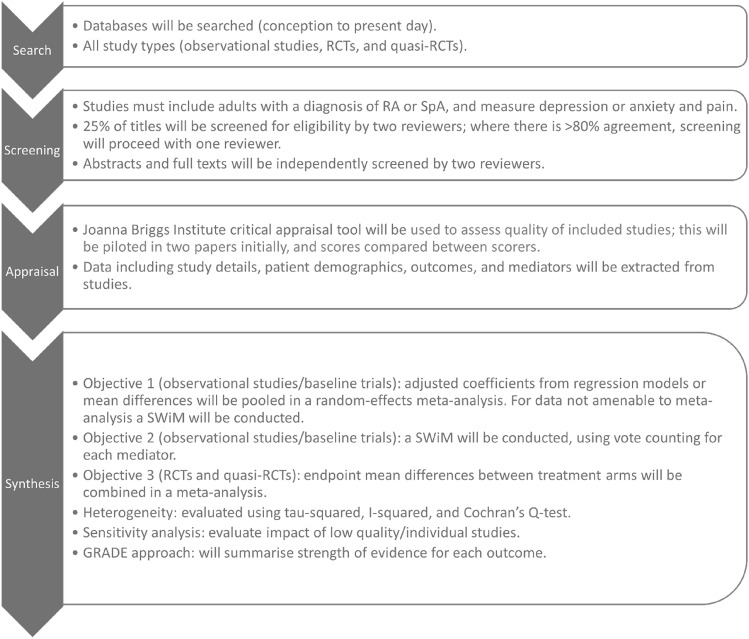
Methodological overview of systematic review. *RCT* randomised controlled trial; *RA*  rheumatoid arthritis; *SpA* spondyloarthritis; *SWiM* synthesis without meta-analysis

The International Prospective Register of Systematic Reviews (PROSPERO; https://www.crd.york.ac.uk/prospero/#aboutpage) was searched (on 1/2/2023) to identify pre-existing protocols related to this review’s aims (none identified). Our protocol was subsequently PROSPERO registered (CRD42023411823). It was prepared using the Preferred Reporting Items for Systematic Reviews and Meta-Analysis Protocols (PRISMA-P) 2015 statement [16]; each stage of the review will comply with the Preferred Reporting Items for Systematic Reviews and Meta-Analyses (PRISMA) 2020 statement (and relevant extensions) [17]. Figure 1 provides a methodological overview of the review.

### Iteratively developed search strategy

A clinical academic (NC) devised the initial strategy (supported by information specialists [acknowledgements]). This was reviewed and refined by two other clinical academics in rheumatology and mental health (ICS and SF), leading to a comprehensive final search strategy (Supplementary Tables 1–5).

Databases searched will comprise MEDLINE and EMBASE (Ovid platform), Cochrane Central Register of Controlled Trials, APA PsychINFO, and Cumulated Index to Nursing and Allied Health Literature plus (conception to present day). Bibliographies of included studies and relevant guidelines from the National Institute for Health and Care Excellence, European Alliance of Associations for Rheumatology, British Society for Rheumatology, and American College of Rheumatology will also be searched.

### Inclusion criteria

These differ depending on the objective.

For objectives 1 and 2, these comprise: (a) observational studies (i.e., cohort, case–control, cross-sectional) or clinical trials (considering baseline data), (b) including adults (aged ≥ 18 years) with a diagnosis of RA and/or SpA, and (c) reporting the relationship between pain and depression/anxiety (with/without details on association mediators). Depression and anxiety will be considered as established diagnoses or the use of validated outcome measures enabling their presence to be determined (e.g., Hospital Anxiety and Depression Scale [HADS] [18].

For objective 3, these comprise: (a) randomised/quasi-randomised controlled trials, (b) including adults (aged ≥ 18 years) with a diagnosis of RA and/or SpA, (c) randomising participants to a pharmacological (anti-depressant/anxiolytic) or psychological (e.g., cognitive behavioural therapy) treatment to manage depression/anxiety, and (d) reporting a pain outcome as an endpoint.

### Exclusion criteria

For all objectives, these comprise: (a) studies in mixed populations where data for patients with RA/SpA cannot be separated from other groups, (b) non-English language studies for which translation cannot be obtained, (c) case reports/series, (d) review articles/editorials, and (e) abstracts/letters without sufficient data for extraction. Additionally, for objective 2, cross-sectional studies will be excluded from the analysis of mediators, and for objective 3, trials that evaluate the efficacy of an intervention at improving pain irrespective of mental health status will be excluded.

### Study screening and selection

Database search results will have duplicates removed and be imported into Rayyan (systematic review management tool) [19]. Retrieved reference titles, abstracts and full texts will be screened for eligibility independently by two reviewers (NC and AH). At all stages, exclusion reasons will be recorded, and disputes resolved through discussion, involving a third reviewer (ICS) where discrepancies remain. Reviewer percentage of agreement will be reported, with Cohen’s kappa coefficient determining interrater reliability.

### Outcome measures

For all objectives, measures of (a) pain intensity (e.g., VAS), (b) other pain dimensions (e.g., high-impact chronic pain), (c) depression (e.g., patient health questionnaire-9 [PHQ-9], (d) anxiety (e.g., HADS), (e) function (e.g., health assessment questionnaire [HAQ]), (f) disease activity (e.g., DAS28), (g) quality of life (e.g., SF-36), (h) other psychological outcomes (e.g., stress), will be considered.

### Data extraction

Two reviewers (NC and AH) will extract data. Separate extraction tables will be used for descriptive data summarising study methods/populations, and data used to address the objectives, which will be initially piloted using two articles. A comprehensive overview of all items to be extracted can be found in the provisional extraction tables (Supplementary Tables 6–9).

### Study quality and risk of bias assessment

The relevant Joanna Briggs Institute critical appraisal tool (dependant on study design) will be used to assess study quality and risk of bias (website reference: https://jbi.global/critical-appraisal-tools). This will be conducted by two authors (NC and AH), with discrepancies resolved through discussion (consulting a third reviewer [ICS] if required). This will be piloted in two papers and scores compared between scorers to ensure standardisation. All studies will then be appraised.

### Data synthesis

This will differ between the objectives and depend on data availability.

#### Objective 1: association between pain and depression/anxiety

Where data for the associations between pain and depression/anxiety are suitable for meta-analysis, the following three steps will be conducted for depression and anxiety in IA subtypes separately. First, for studies evaluating the association between pain and depression/anxiety using regression models, adjusted coefficient values will be pooled using a random effects model with 95% confidence intervals (CIs), applying DerSimonian and Laird’s method [20], with *P* values used where standard errors (SEs) are not reported. Second, for studies reporting mean pain scores by depression/anxiety categories, in studies using the same pain scale of measurement (e.g., pain intensity VAS) mean pain scores and standard deviations (SDs) will be extracted for each depression/anxiety category and effect estimates of depression/anxiety on pain calculated using the mean difference (MD) in these scores and combined using a random-effects model with 95% CIs. Third, for studies using different pain measurement scales (e.g., VAS and SF-36 bodily pain) the effect estimates of depression/anxiety on pain will be calculated using the standardised mean difference (SMD). Steps two and three will be repeated looking at effect estimates of pain on depression/anxiety.

Where insufficient data are available for meta-analysis, a synthesis without meta-analysis (SWiM) will be conducted for depression and anxiety separately. First studies will be grouped by IA subtypes. Subsequently, for each study summary statistics for the relationship between pain and depression/anxiety (and any *P* values for tests evaluating statistical significance) will be extracted, with *P* values combined where possible or vote counting based on direction of effect undertaken.

#### Objective 2: mediators of the relationship between pain and depression/anxiety

Due to an anticipated lack studies reporting on this outcome, a SWiM (conducted as for objective 1) is planned to summarise the variables mediating any associations between pain and depression/anxiety.

#### Objective 3: Impact of treating co-morbid depression/anxiety on pain

Where data for the impact of treating depression/anxiety on pain are suitable for meta-analysis, the following three steps will be conducted separately for depression and anxiety, IA subtypes, and treatment modality (e.g., pharmacological/psychological).

First, in studies using the same pain outcome scale, mean (and SD) endpoint scores will be extracted for treatment arms, and effect estimates of treatment for depression on pain calculated using the MD and combined using a random effects model with 95% CIs. Second, in studies using different outcome measures to assess the same pain construct, step one will be repeated using SMD in lieu of MD. Third, in studies reporting binary outcomes (e.g., ≥ 50% improvement in pain intensity scores or not), pooled odds ratios (ORs) will be calculated with 95% CIs using random-effects models.

### Heterogeneity

Where possible, statistical heterogeneity will be summarised using the estimate of between study variance (tau-squared), and the proportion of variability in effect estimates due to between study heterogeneity (I-squared), and Cochran’s Q-test (*p* < 0.1 will be considered significant heterogeneity).

### Publication bias

This will be visually assessed by a funnel plot and its asymmetry tested by Egger’s method if ≥ 10 studies are available for a given comparison. The trim-and-fill method to estimate the summary effect size will be applied if there is no evidence of publication bias [21].

### Subgroup and sensitivity analysis

Where possible, a subgroup analysis will describe the efficacy of different treatment modalities (e.g., anti-depressant classes, psychological intervention type), and a sensitivity analysis will examine the impact of low-quality studies and removing individual studies.

### Strength of evidence

Two reviewers will judge the strength of evidence for each outcome and present findings in a “Summary of Findings Table” using the Grading of Recommendations Assessment, Development and Evaluation approach [22].

## Discussion

Pain is a major concern of patients with IA. In large, internationally conducted patient surveys, approximately two-thirds of patients with RA report dissatisfaction with arthritis pain [1], over 80% of patients with PsA report pain in the past year [23], and 31% of patients with axial SpA state “suffering pain” is a common disease-related fear [24]. Despite receiving high-cost biologic drugs, 79% of patients with RA in the British Society for Rheumatology Biologics Registry have persistent pain [25]. Understanding how to best assess and manage pain in IA is, therefore, an important clinical and research goal. Our planned systematic review will support this from the perspective of mental health. Through better defining the relationship between depression/anxiety and pain in patients with IA—spanning the strength and direction of any associations and their potential mediators, alongside the impact of treating depression and anxiety on pain—our review has the potential to inform the development and implementation of mental health assessment and management processes when treating pain in patients with IA.

The planned systematic review’s strengths are it: (1) complies with recommended PRISMA frameworks, (2) has been iteratively developed with an experienced team of researchers, (3) has a peer-reviewed search strategy, and (4) has a pre-defined, rigorous synthesis plan for each objective, which focuses on statistical approaches. Its limitations include: (1) due to anticipated heterogeneity in the way studies will measure and assess mediators, a SWiM will be conducted for objective 2, and (2) it will focus on the relationship between pain and depression/anxiety, and the impact of other drivers of pain in IA (e.g., disease activity) will not be directly considered.

### Supplementary Information

Below is the link to the electronic supplementary material.

Supplementary file1 (XLSX 28 KB)

Supplementary file2 (DOCX 45 KB)
