# Supplementary material for: The relationship between pain and depression and anxiety in patients with inflammatory arthritis: a systematic review protocol
Source: Rheumatol Int. 2023 Sep 12;44(3):435–40. doi: 10.1007/s00296-023-05450-y (PMC10867049; doi:10.1007/s00296-023-05450-y)
Supplement: Supplementary file 2 — Supplementary file2 (DOCX 45 KB) [file 296_2023_5450_MOESM2_ESM.docx]

**The Relationship Between Pain and Depression and Anxiety in Patients with Inflammatory Arthritis: A Systematic Review Protocol**

**Supplementary Material**

Natasha Cox, Ashley Hawarden, Ram Bajpai, Saeed Farooq, Helen Twohig, Sara Muller, Ian C Scott.

**Supplementary Table 1. Search Strategy MEDLINE (OVID)**

| 1 | exp arthritis, rheumatoid/ |
| --- | --- |
| 2 | ((rheumato*) adj3 (arthrit* or diseas* or condition* or nodule*)).ti,ab,kf. |
| 3 | (felty* adj2 syndrome). ti,ab,kf. |
| 4 | (caplan* adj2 syndrome). ti,ab,kf. |
| 5 | or/1‐4 |
| 6 | exp Arthritis, psoriatic/ |
| 7 | (psoria* adj3 (arthrit* or arthropathy* or polyarthr* or enthesitis or polyarthr)). ti,ab,kf. |
| 8 | psoriasis/ and exp arthritis/ |
| 9 | (arthri* adj2 mutilans).ti,ab,kf. |
| 10 | or/6-9 |
| 11 | (axial adj3 (PSA or psoria*)). ti,ab,kf. |
| 12 | (psoria* adj3 (axial SpA or axSpA or spondylitis )). ti,ab,kf. |
| 13 | or/11-12 |
| 14 | (ankylosing ADJ spondyl*). ti,ab,kf. |
| 15 | exp Spondylitis, ankylosing |
| 16 | exp spondyloarthritis, axial/ |
| 17 | Bechtere*. ti,ab,kf. |
| 18 | Marie-str*. ti,ab,kf. |
| 19 | (Bamboo ADJ spine). ti,ab,kf. |
| 20 | Sacroiliit*. ti,ab,kf. |
| 21 | exp Sacroiliitis |
| 22 | or/ 14-21 |
| 23 | Spondyloarth*. ti,ab,kf. |
| 24 | Spondylarth*. ti,ab,kf. |
| 25 | exp Spondylarthritis |
| 26 | exp Spondylarthropathies |
| 27 | or/23-26 |
| 28 | exp arthritis, reactive/ |
| 29 | (reactive adj3 arthr*).ti,ab,kf. |
| 30 | reiter*.ti,ab,kf. |
| 31 | or/ 28-30 |
| 32 | (entero* or bowel* or chron* or colitis* or ulcerative* gastro*) adj4 (arthr* or spondyl*).ti,ab,kf. |
| 33 | exp Depression/ |
| 34 | exp depressive disorder/ |
| 35 | exp depressive disorder, major/ |
| 36 | exp mood disorders/ |
| 37 | exp dysthymic disorder/ |
| 38 | depres*. ti,ab,kf. |
| 39 | (depress* adj illness). ti,ab,kf. |
| 40 | (mood adj4 disorder*). ti,ab,kf. |
| 41 | dysthymi*. ti,ab,kf. |
| 42 | or/ 33-41 |
| 43 | exp anxiety/ |
| 44 | Anxi*. ti,ab,kf. |
| 45 | exp anxiety disorders/ |
| 46 | (anxi* adj3 dis*).ti,ab,kf. |
| 47 | (general* adj anxiety adj disorder*).ti,ab,kf. |
| 48 | (panic adj disorder*).ti,ab,kf. |
| 49 | or/ 43-48 |
| 50 | (common* adj2 mental* adj health adj disorder*).ti,ab,kf. |
| 51 | 5 OR 10 OR 13 OR 22 OR 27 OR 31 OR 32 |
| 52 | 42 OR 49 OR 50 |
| 53 | 51 AND 52 |

**Supplementary Table 2. Search Strategy EMBASE (OVID)**

| 1 | exp arthritis, rheumatoid/ |
| --- | --- |
| 2 | ((rheumato*) adj3 (arthrit* or diseas* or condition* or nodule*)).ti,ab,kf. |
| 3 | (felty* adj2 syndrome). ti,ab,kf. |
| 4 | (caplan* adj2 syndrome). ti,ab,kf. |
| 5 | or/1‐4 |
| 6 | exp Arthritis, psoriatic/ |
| 7 | (psoria* adj3 (arthrit* or arthropathy* or polyarthr* or enthesitis or polyarthr)). ti,ab,kf. |
| 8 | psoriasis/ and exp arthritis/ |
| 9 | (arthri* adj2 mutilans).ti,ab,kf. |
| 10 | or/6-9 |
| 11 | (axial adj3 (PSA or psoria*)). ti,ab,kf. |
| 12 | (psoria* adj3 (axial SpA or axSpA or spondylitis )). ti,ab,kf. |
| 13 | or/11-12 |
| 14 | (ankylosing ADJ spondyl*). ti,ab,kf. |
| 15 | exp Spondylitis, ankylosing |
| 16 | exp spondyloarthritis, axial/ |
| 17 | Bechtere*. ti,ab,kf. |
| 18 | Marie-str*. ti,ab,kf. |
| 19 | (Bamboo ADJ spine). ti,ab,kf. |
| 20 | Sacroiliit*. ti,ab,kf. |
| 21 | exp Sacroiliitis |
| 22 | or/ 14-21 |
| 23 | Spondyloarth*. ti,ab,kf. |
| 24 | Spondylarth*. ti,ab,kf. |
| 25 | exp Spondylarthritis |
| 26 | exp Spondylarthropathies |
| 27 | or/23-26 |
| 28 | exp arthritis, reactive/ |
| 29 | (reactive adj3 arthr*).ti,ab,kf. |
| 30 | reiter*.ti,ab,kf. |
| 31 | or/ 28-30 |
| 32 | (entero* or bowel* or chron* or colitis* or ulcerative* gastro*) adj4 (arthr* or spondyl*).ti,ab,kf. |
| 33 | exp Depression/ |
| 34 | exp depressive disorder/ |
| 35 | exp depressive disorder, major/ |
| 36 | exp mood disorders/ |
| 37 | exp dysthymic disorder/ |
| 38 | depres*. ti,ab,kf. |
| 39 | (depress* adj illness). ti,ab,kf. |
| 40 | (mood adj4 disorder*). ti,ab,kf. |
| 41 | dysthymi*. ti,ab,kf. |
| 42 | or/ 33-41 |
| 43 | exp anxiety/ |
| 44 | Anxi*. ti,ab,kf. |
| 45 | exp anxiety disorders/ |
| 46 | (anxi* adj3 dis*).ti,ab,kf. |
| 47 | (general* adj anxiety adj disorder*).ti,ab,kf. |
| 48 | (panic adj disorder*).ti,ab,kf. |
| 49 | or/ 43-48 |
| 50 | (common* adj2 mental* adj health adj disorder*).ti,ab,kf. |
| 51 | 5 OR 10 OR 13 OR 22 OR 27 OR 31 OR 32 |
| 52 | 42 OR 49 OR 50 |
| 53 | 51 AND 52 |
| 54 | limit 53 to embase only |

**Supplementary Table 3. Search Strategy CLINAHL Plus (EBSCO)**

| 1 | (MH “rheumatoid arthritis+”) |
| --- | --- |
| 2 | TI ( ((rheumato*) W3 (arthrit* or diseas* or condition* or nodule*)) ) OR AB ( ((rheumato*) W3 (arthrit* or diseas* or condition* or nodule*)) ) OR KW ( ((rheumato*) W3 (arthrit* or diseas* or condition* or nodule*)) ) |
| 3 | TI (felty* W2 syndrome) OR AB (felty* W2 syndrome) OR KW (felty* W2 syndrome) |
| 4 | TI (caplan* W2 syndrome) OR AB (caplan* W2 syndrome) OR KW (caplan* W2 syndrome) |
| 5 | or/1‐4 |
| 6 | (MH “arthritis psoriatic+”) |
| 7 | TI ( (psoria* W3 (arthrit* or arthropathy* or polyarthr* or enthesitis or polyarthr)) ) OR AB ( (psoria* W3 (arthrit* or arthropathy* or polyarthr* or enthesitis or polyarthr)) ) OR KW ( (psoria* W3 (arthrit* or arthropathy* or polyarthr* or enthesitis or polyarthr)) ) |
| 8 | (DE “psoriasis”) AND (MH arthritis+) |
| 9 | TI mutilans OR AB mutilans OR KW mutilans |
| 10 | or/6-9 |
| 11 | TI ( (axial W3 (PSA or psoria*)) ) OR AB ( (axial W3 (PSA or psoria*)) ) OR KW ( (axial W3 (PSA or psoria*)) ) |
| 12 | TI ( (psoria* W3 (axial SpA or axSpA or spondylitis)) ) OR AB ( (psoria* W3 (axial SpA or axSpA or spondylitis)) ) OR KW ( (psoria* W3 (axial SpA or axSpA or spondylitis)) ) |
| 13 | or/11-12 |
| 14 | TI (ankylosing W1 spondyl*) OR AB (ankylosing W1 spondyl*) OR KW (ankylosing W1 spondyl*) |
| 15 | (MH “ankylosing spondylitis+”) |
| 16 | (MH “axial spondyloarthritis+”) |
| 17 | TI Bechtere* OR AB Bechtere* OR KW Bechtere* |
| 18 | TI Marie-str* OR AB Marie-str* OR KW Marie-str* |
| 19 | TI (Bamboo W1 spine) OR AB (Bamboo W1 spine) OR KW (Bamboo W1 spine) |
| 20 | TI Sacroiliit* OR AB Sacroiliit* OR KW Sacroiliit* |
| 21 | (MH “sacroiliitis+”) |
| 22 | or/ 14-21 |
| 23 | TI Spondyloarth* OR AB Spondyloarth* OR KW Spondyloarth* |
| 24 | TI Spondylarth* OR AB Spondylarth* OR KW Spondylarth* |
| 25 | (MH “spondylarthritis+”) |
| 26 | (MH “spondyloarthropathies+”) |
| 27 | or/23-26 |
| 28 | (MH “reactive arthritis+”) |
| 29 | TI (reactive W3 arthr*) OR AB (reactive W3 arthr*) OR KW (reactive W3 arthr*) |
| 30 | TI reiter* OR AB reiter* OR KW reiter* |
| 31 | or/ 28-30 |
| 32 | TI ( (entero* or bowel* or chron* or colitis* or ulcerative* gastro*) W4 (arthr* or spondyl*) ) OR AB ( (entero* or bowel* or chron* or colitis* or ulcerative* gastro*) W4 (arthr* or spondyl*) ) OR KW ( (entero* or bowel* or chron* or colitis* or ulcerative* gastro*) W4 (arthr* or spondyl*) ) |
| 33 | (MH "Depression+") |
| 34 | (MH " Depressive Disorder+") |
| 35 | (MH " Depressive Disorder, major+") |
| 36 | (MH “mood disorder+”) |
| 37 | (MH "Dysthymic Disorder+") |
| 38 | TI depres* OR AB depres* OR KW depres* |
| 39 | TI (depress* W1 illness) OR AB (depress* W1 illness) OR KW (depress* W1 illness) |
| 40 | TI (mood W4 disorder*) OR AB (mood W4 disorder*) OR KW (mood W4 disorder*) |
| 41 | TI dysthymi* OR AB dysthymi* OR KW dysthymi* |
| 42 | or/ 33-41 |
| 43 | (MH “anxiety+”) |
| 44 | TI Anxi* OR AB Anxi* OR KW Anxi* |
| 45 | (MH “anxiety disorder+”) |
| 46 | TI (anxi* W3 dis*) OR AB (anxi* W3 dis*) OR KW (anxi* W3 dis*) |
| 47 | TI (general* W1 anxiety W1 disorder*) OR AB (general* W1 anxiety W1 disorder*) OR KW (general* W1 anxiety W1 disorder*) |
| 48 | TI (panic W1 disorder*) OR AB (panic W1 disorder*) OR KW (panic W1 disorder*) |
| 49 | or/ 43-48 |
| 50 | TI ((common* W2 mental* W1 health W1 disorder*)) OR AB ((common* W2 mental* W1 health W1 disorder*)) OR KW ((common* W2 mental* W1 health W1 disorder*)) |
| 51 | 5 OR 10 OR 13 OR 22 OR 27 OR 31 OR 32 |
| 52 | 42 OR 49 OR 50 |
| 53 | 51 AND 52 |

**Supplementary Table 4. Search Strategy psycINFO (EBSCO)**

| 1 | DE "Rheumatoid Arthritis" |
| --- | --- |
| 2 | TI ( ((rheumato*) W3 (arthrit* or diseas* or condition* or nodule*)) ) OR AB ( ((rheumato*) W3 (arthrit* or diseas* or condition* or nodule*)) ) OR KW ( ((rheumato*) W3 (arthrit* or diseas* or condition* or nodule*)) ) |
| 3 | TI (felty* W2 syndrome) OR AB (felty* W2 syndrome) OR KW (felty* W2 syndrome) |
| 4 | TI (caplan* W2 syndrome) OR AB (caplan* W2 syndrome) OR KW (caplan* W2 syndrome) |
| 5 | or/1‐4 |
| 6 | MA arthritis psoriatic |
| 7 | TI ( (psoria* W3 (arthrit* or arthropathy* or polyarthr* or enthesitis or polyarthr)) ) OR AB ( (psoria* W3 (arthrit* or arthropathy* or polyarthr* or enthesitis or polyarthr)) ) OR KW ( (psoria* W3 (arthrit* or arthropathy* or polyarthr* or enthesitis or polyarthr)) ) |
| 8 | MA psoriasis AND (MH arthritis+) |
| 9 | TI mutilans OR AB mutilans OR KW mutilans |
| 10 | or/6-9 |
| 11 | TI ( (axial W3 (PSA or psoria*)) ) OR AB ( (axial W3 (PSA or psoria*)) ) OR KW ( (axial W3 (PSA or psoria*)) ) |
| 12 | TI ( (psoria* W3 (axial SpA or axSpA or spondylitis)) ) OR AB ( (psoria* W3 (axial SpA or axSpA or spondylitis)) ) OR KW ( (psoria* W3 (axial SpA or axSpA or spondylitis)) ) |
| 13 | or/11-12 |
| 14 | TI (ankylosing W1 spondyl*) OR AB (ankylosing W1 spondyl*) OR KW (ankylosing W1 spondyl*) |
| 15 | MA ankylosing spondylitis |
| 16 | MA axial spondyloarthritis |
| 17 | TI Bechtere* OR AB Bechtere* OR KW Bechtere* |
| 18 | TI Marie-str* OR AB Marie-str* OR KW Marie-str* |
| 19 | TI (Bamboo W1 spine) OR AB (Bamboo W1 spine) OR KW (Bamboo W1 spine) |
| 20 | TI Sacroiliit* OR AB Sacroiliit* OR KW Sacroiliit* |
| 21 | MA sacroiliitis |
| 22 | or/ 14-21 |
| 23 | TI Spondyloarth* OR AB Spondyloarth* OR KW Spondyloarth* |
| 24 | TI Spondylarth* OR AB Spondylarth* OR KW Spondylarth* |
| 25 | MA spondylarthritis |
| 26 | MA spondyloarthropathies |
| 27 | or/23-26 |
| 28 | MA reactive arthritis |
| 29 | TI (reactive W3 arthr*) OR AB (reactive W3 arthr*) OR KW (reactive W3 arthr*) |
| 30 | TI reiter* OR AB reiter* OR KW reiter* |
| 31 | or/ 28-30 |
| 32 | TI ( (entero* or bowel* or chron* or colitis* or ulcerative* gastro*) W4 (arthr* or spondyl*) ) OR AB ( (entero* or bowel* or chron* or colitis* or ulcerative* gastro*) W4 (arthr* or spondyl*) ) OR KW ( (entero* or bowel* or chron* or colitis* or ulcerative* gastro*) W4 (arthr* or spondyl*) ) |
| 33 | DE "Depression (Emotion)" |
| 34 | DE "Persistent Depressive Disorder" |
| 35 | DE "Major Depression" OR DE "Anaclitic Depression" OR DE "Dysthymic Disorder" OR DE "Endogenous Depression" OR DE "Late Life Depression" OR DE "Postpartum Depression" OR DE "Reactive Depression" OR DE "Recurrent Depression" OR DE "Treatment Resistant Depression" |
| 36 | DE "Affective Disorders" OR DE "Disruptive Mood Dysregulation Disorder" OR DE "Major Depression" OR DE "Persistent Depressive Disorder" OR DE "Premenstrual Dysphoric Disorder" OR DE "Seasonal Affective Disorder" |
| 37 | DE "Dysthymic Disorder" |
| 38 | TI depres* OR AB depres* OR KW depres* |
| 39 | TI (depress* W1 illness) OR AB (depress* W1 illness) OR KW (depress* W1 illness) |
| 40 | TI (mood W4 disorder*) OR AB (mood W4 disorder*) OR KW (mood W4 disorder*) |
| 41 | TI dysthymi* OR AB dysthymi* OR KW dysthymi* |
| 42 | or/ 33-41 |
| 43 | DE "Anxiety" OR DE "Anxiety Sensitivity" OR DE "Climate Anxiety" OR DE "Computer Anxiety" OR DE "Death Anxiety" OR DE "Health Anxiety" OR DE "Mathematics Anxiety" OR DE "Performance Anxiety" OR DE "Social Anxiety" OR DE "Speech Anxiety" OR DE "Test Anxiety" OR DE "Travel Anxiety" |
| 44 | TI Anxi* OR AB Anxi* OR KW Anxi* |
| 45 | DE "Anxiety Disorders" OR DE "Castration Anxiety" OR DE "Generalized Anxiety Disorder" OR DE "Panic Attack" OR DE "Panic Disorder" OR DE "Phobias" OR DE "Selective Mutism" OR DE "Separation Anxiety Disorder" |
| 46 | TI (anxi* W3 dis*) OR AB (anxi* W3 dis*) OR KW (anxi* W3 dis*) |
| 47 | TI (general* W1 anxiety W1 disorder*) OR AB (general* W1 anxiety W1 disorder*) OR KW (general* W1 anxiety W1 disorder*) |
| 48 | TI (panic W1 disorder*) OR AB (panic W1 disorder*) OR KW (panic W1 disorder*) |
| 49 | or/ 43-48 |
| 50 | TI ((common* W2 mental* W1 health W1 disorder*)) OR AB ((common* W2 mental* W1 health W1 disorder*)) OR KW ((common* W2 mental* W1 health W1 disorder*)) |
| 51 | 5 OR 10 OR 13 OR 22 OR 27 OR 31 OR 32 |
| 52 | 42 OR 49 OR 50 |
| 53 | 51 AND 52 |

**Supplementary Table 5. Search Strategy Cochrane Central Register of Controlled Trials (CENTRAL)**

| 1 | MeSH descriptor: [Arthritis, Rheumatoid] explode all trees |
| --- | --- |
| 2 | (((rheumato*) NEXT/3 (arthrit* or diseas* or condition* or nodule*))):ti,ab,kw |
| 3 | ((felty* NEXT/2 syndrome)):ti,ab,kw |
| 4 | ((caplan*) NEXT/2 (syndrome)):ti,ab,kw (Word variations have been searched) |
| 5 | or/1‐4 |
| 6 | MeSH descriptor: [Arthritis, Psoriatic] explode all trees |
| 7 | ((psoria* NEXT/3 (arthrit* or arthropathy* or polyarthr* or enthesitis or polyarthr*))):ti,ab,kw |
| 8 | MeSH descriptor: [Psoriasis] this term only |
| 9 | MeSH descriptor: [Arthritis] explode all trees |
| 10 | 8 AND 9 |
| 11 | ((arthri* NEXT/2 mutilans)):ti,ab,kw |
| 12 | 6 OR 7 OR 10 OR 11 |
| 13 | ((axial NEXT/3 (PSA or psoria*))):ti,ab,kw |
| 14 | ((psoria* NEXT/3 (axial SpA or axSpA or spondylitis))):ti,ab,kw |
| 15 | or/ 13-14 |
| 16 | ((ankylosing NEXT spondyl*)):ti,ab,kw |
| 17 | MeSH descriptor: [Spondylitis, Ankylosing] explode all trees |
| 18 | MeSH descriptor: [Axial Spondyloarthritis] explode all trees |
| 19 | (Bechtere*):ti,ab,kw |
| 20 | (Marie-str*):ti,ab,kw |
| 21 | ((Bamboo NEXT spine)):ti,ab,kw |
| 22 | (Sacroiliit*):ti,ab,kw |
| 23 | MeSH descriptor: [Sacroiliitis] explode all trees |
| 24 | or/16-23 |
| 25 | (Spondyloarth*):ti,ab,kw |
| 26 | (Spondylarth*):ti,ab,kw |
| 27 | MeSH descriptor: [Spondylarthritis] explode all trees |
| 28 | MeSH descriptor: [Spondylarthropathies] explode all trees |
| 29 | or/25-28 |
| 30 | MeSH descriptor: [Arthritis, Reactive] explode all trees |
| 31 | ((reactive NEXT/3 arthr*)):ti,ab,kw |
| 32 | (reiter*):ti,ab,kw |
| 33 | or/30-32 |
| 34 | ((entero* or bowel* or chron* or colitis* or ulcerative* gastro*) NEXT/4 (arthr* or spondyl*)):ti,ab,kw |
| 35 | MeSH descriptor: [Depression] explode all trees |
| 36 | MeSH descriptor: [Depressive Disorder] explode all trees |
| 37 | MeSH descriptor: [Depressive Disorder, Major] explode all trees |
| 38 | MeSH descriptor: [Mood Disorders] explode all trees |
| 39 | MeSH descriptor: [Dysthymic Disorder] explode all trees |
| 40 | (depres*):ti,ab,kw |
| 41 | ((depress* NEXT illness)):ti,ab,kw |
| 42 | ((mood NEXT/4 disorder*)):ti,ab,kw |
| 43 | (dysthymi*):ti,ab,kw |
| 44 | or/35-43 |
| 45 | MeSH descriptor: [Anxiety] explode all trees |
| 46 | (anxi*):ti,ab,kw |
| 47 | MeSH descriptor: [Anxiety Disorders] explode all trees |
| 48 | ((anxi* NEXT/3 dis*)):ti,ab,kw |
| 49 | ((general* NEXT anxiety NEXT disorder*)):ti,ab,kw |
| 50 | ((panic NEXT disorder*)):ti,ab,kw |
| 51 | or/45-50 |
| 52 | ((common* NEXT/2 mental* NEXT health NEXT disorder*)):ti,ab,kw |
| 53 | 5 OR 12 OR 15 OR 24 OR 29 OR 33 OR 34 |
| 54 | 44 OR 51 OR 52 |
| 55 | 53 AND 54 |
